# Skinny kelp (*Saccharina angustissima*) provides valuable genetics for the biomass improvement of farmed sugar kelp (*Saccharina latissima*)

**DOI:** 10.1007/s10811-022-02811-1

**Published:** 2022-08-20

**Authors:** Yaoguang Li, Schery Umanzor, Crystal Ng, Mao Huang, Michael Marty-Rivera, David Bailey, Margaret Aydlett, Jean-Luc Jannink, Scott Lindell, Charles Yarish

**Affiliations:** 1 Department of Ecology & Evolutionary Biology, University of Connecticut, Stamford, CT 06901-2315 USA; 2 College of Fisheries and Ocean Sciences, University of Alaska Fairbanks, Juneau, AK 99775 USA; 3 Section On Plant Breeding and Genetics, School of Integrative Plant Sciences, Cornell University, Ithaca, NY 14853 USA; 4 Applied Ocean Physics and Engineering, Woods Hole Oceanographic Institution, Woods Hole, MA 02543 USA; 5 United States Department of Agriculture - Agriculture Research Service, Ithaca, NY 14853 USA

**Keywords:** *Saccharina latissima*, *Saccharina angustissima*, Morphological trait, Biomass, Seaweed aquaculture

## Abstract

*Saccharina latissima* (sugar kelp) is one of the most widely cultivated brown marine macroalgae species in the North Atlantic and the eastern North Pacific Oceans. To meet the expanding demands of the sugar kelp mariculture industry, selecting and breeding sugar kelp that is best suited to offshore farm environments is becoming necessary. To that end, a multi-year, multi-institutional breeding program was established by the U.S. Department of Energy's (DOE) Advanced Research Projects Agency-Energy (ARPA-E) Macroalgae Research Inspiring Novel Energy Resources (MARINER) program. Hybrid sporophytes were generated using 203 unique gametophyte cultures derived from wild-collected *Saccharina* spp*.* for two seasons of farm trials (2019–2020 and 2020–2021). The wild sporophytes were collected from 10 different locations within the Gulf of Maine (USA) region, including both sugar kelp (*Saccharina latissima*) and the skinny kelp species (*Saccharina angustissima*). We harvested 232 common farm plots during these two seasons with available data. We found that farmed kelp plots with skinny kelp as parents had an average increased yield over the mean (wet weight 2.48 ± 0.90 kg m^−1^ and dry weight 0.32 ± 0.10 kg m^−1^) in both growing seasons. We also found that blade length positively correlated with biomass in skinny kelp x sugar kelp crosses or pure sugar kelp crosses. The skinny x sugar progenies had significantly longer and narrower blades than the pure sugar kelp progenies in both seasons. Overall, these findings suggest that sugar x skinny kelp crosses provide improved yield compared to pure sugar kelp crosses.

**Supplementary Information:**

The online version contains supplementary material available at 10.1007/s10811-022-02811-1.

## Introduction

Seaweed has been widely harvested and used for hundreds of years worldwide (Buschmann et al. 2017; Kim et al. 2017; Mouritsen et al. 2018). Since the 1960s global seaweed production has shifted from wild harvesting to cultivation, with a total global production of 35.8 million tonnes wet biomass, accounting for 97 percent of the world seaweed production in 2019 (Cai 2021). Brown seaweed comprises a large portion of seaweed production, with 35.4 percent of the world's cultivated seaweed biomass (Cai 2021). Seaweed can be used in animal feed, cosmetics, human food, fertilizer, and biofuels (Wargacki et al. 2012; Peteiro et al. 2014; Rajauria 2015; Michalak 2018). In 2019, the USA alone exported 18 million US$ and imported 95 million US$ of seaweed (Cai 2021). As the demand for kelp or kelp-related products increases rapidly in the U.S. and globally, U.S. kelp farms are poised to expand to meet the needs of the seaweed industry.

The rapid increase of seaweed production since the 1950s can be attributed to improved industrialized large-scale cultivation methods, such as the summer seedling-raising method, floating raft culture systems for *Saccharina japonica* in China (Tseng et al. 1955; Tseng 2001), and hatchery propagation of nori in Japan (Miura 1980; Yarish and Pereira 2008). In parallel to the improvement of cultivation methods, breeding programs developed advanced lines for improving seaweed yield and phenotype (Shan et al. 2013; Hwang et al. 2019; Hu et al. 2021). Seaweed breeding programs are well developed, especially in Asian countries, including China, Japan and South Korea, and recently seaweed breeding has gained attention in other parts of the world, such as Europe, North and South America (Camus et al. 2019; Hwang et al. 2019; Visch 2019; Wang et al. 2020; Huang et al. 2022). In Asian countries, generations of advanced cultivars have been developed, registered by government authorities, and cultivated by local farms (Hwang et al. 2019; Wang et al. 2020). To generate these advanced cultivars, diverse breeding strategies have been applied. These include traditional selective breeding, inter- and intra- species hybridization, and marker assisted selection. The target traits include, but are not limited to, high biomass, large blades, disease resistance, and high-temperature tolerance. (Li et al. 2007, 2008, 2016a, b; Zhang et al. 2007; Cui et al. 2017).

Sugar kelp, *Saccharina latissima* (Linnaeus) C.E. Lane, C. Mayes, Druehl & G.W. Saunders, is a brown macroalga whose southern distribution in the western North Atlantic region is Long Island Sound (40.7605–41.4221° N, 71.8383–73.9390° W) (Egan and Yarish 1988; Yarish and Egan 1989; Yarish et al. 1990). Due to its relatively high sugar content, sugar kelp is also considered a potential candidate for biofuel in the U.S. and Northern Europe (Yarish et al. 2017; Bak et al. 2018; Kerrison et al. 2018; Kim et al. 2019). The Gulf of Maine region has extensive natural beds of *S. latissima*. A closely related species, *Saccharina angustissima* (Collins) Augyte, Yarish, & Neefus (also known as skinny kelp) has a restricted natural distribution. *S. angustissima* is characterized by having a thick, narrow, long strap-like blade morphology and grows in the low intertidal zone in Casco Bay in southern Maine (43.7230°N, 69.9942°W). The unique morphology of *S. angustissima* may be attributed to an adaptation to low intertidal wave-exposed habitats in the Gulf of Maine (Mathieson et al. 2008; Augyte et al. 2018). Despite the taxonomic separation, *S. angustissima* and *S. latissima* are so closely related that there is no clear evidence of genetic separation based on single nucleotide polymorphic markers. In fact, Mao et al. (2020) found that the two species were less genetically differentiated than were populations of *S. latissima* from the Gulf of Maine and southern New England. In addition, the two species of *Saccharina* are interfertile (Augyte et al. 2017, 2018; Mao et al. 2020; Umanzor et al. 2021). *Saccharina angustissima* has unique culinary and commercial applications, and consistently produces high yields on long-line kelp farms, so breeding this unique kelp will be a crucial part of the future of kelp farming in the Gulf of Maine (Umanzor et al. 2021).

Commercial wild kelp harvests in Maine supply food product sales as well as kelp hatcheries requiring fertile tissues to produce "seed" for kelp farms. Expanding demands for reproductive kelp individuals can negatively impact natural populations, leading to shortages and genetic diversity reduction of wild specimens (J. Robidoux, personal communication). To satisfy the growing demand for kelp and protect wild populations from over-exploitation, two options are available: 1) increase the number and size of kelp farms and/or 2) improve productivity on existing farms. These options are not mutually exclusive, and a combination of both should be employed to produce more biomass. Phycologists, geneticists, and breeders at U.S. research institutions, in cooperation with kelp farmers, are working together to address the second option through two kelp selective breeding programs: sugar kelp in New England and giant kelp (*Macrocystis pyrifera*) in California. These selective breeding programs were initiated in 2018 and supported by the U.S. Department of Energy Advanced Research Projects Agency-Energy (ARPA-E) Macroalgae Research Inspiring Novel Energy Resources (MARINER). The aim of the program is to develop and implement genetic tools to accelerate breeding for high biomass kelp strains thus enabling cost-efficient seaweed farming. Breeding high-yielding cultivars will involve the hybridization of different *S. latissima* strains and hybridization with the closely related *S. angustissima* in the Gulf of Maine for the New England program.

Through our breeding project we invariably observed morphological differences and higher yield with crosses that included *S. angustissima* (skinny kelp) as one of the parents over two growing seasons. These provided information and direction for future kelp breeding in the Gulf of Maine.

## Materials and methods

### Collection of parental sporophytes, spore release, and settlement

Gametophytes used in this experiment were derived from 69 parental sporophytes collected from 10 different locations within the Gulf of Maine, Northwest Atlantic Ocean, from April to December 2018 (Supplement Figure 1). Morphological traits of collected sporophytes were measured including blade length, blade maximum width, blade thickness, stipe length, and stipe diameter (Umanzor et al. 2021). Of the 10 collecting locations, one included a farm site (Casco Bay, Bangs Island Mussels, ME, USA) which was also the source of the skinny kelp, *Saccharina angustissima*.

After measuring all morphological traits of wild collected parental sporophytes, one or two pieces of sorus tissues were excised from each collected sporophyte. Each sorus tissue fragment was cleaned thoroughly by wiping with iodine solution, washed with sterile seawater, and stored overnight at 10 °C in darkness (Redmond et al. 2014). Meiospores from each sporophyte's sorus tissue fragment were then released into corresponding individual sterile seawater beakers the following day. Meiospores were either manually isolated within individual Petri dishes (Redmond et al. 2014) or sorted automatically (*N* = 96 isolations per parental tissue) by a flow cytometer (Bay Bioscience, Japan; Augyte et al. 2020). In the case of the manual isolations, the solution was added to multi-well sterile culture dishes with glass fragments and allowed to settle at 10 °C for 24 h under low light (50 μmol photons m^‑2^ s^‑1^). After the settlement, glass fragments were removed, rinsed with a sterilized seawater stream, and placed in clean Petri dishes with half-strength Provasoli's Enrichment Medium (10 mL PES L^−1^ natural seawater; Provasoli 1968). The dishes were sealed with Parafilm to prevent evaporation.

The gametophyte cultivation and maintenance methods were similar to those described in an earlier study (Umanzor et al. 2021). After settlement, gametophytes were cultured under red light provided by LED bars (Pure Biomass, Minneapolis, MN, USA) or red Plexiglas boxes with a photon fluence rate of 30–60 μmol photons m^‑2^ s^‑1^ for approximately two weeks until male and female filaments could be easily distinguished under a microscope at 10X magnification. After 10–14 days, gametophytes were individually isolated under a dissecting scope using Pasteur pipettes (Fisher Scientific, USA) and isolated filaments were grown into large clonal masses, where each cell was genetically identical.

### Gametophyte cultivation and maintenance

Male and female gametophytes were sexed and labeled on each Petri dish. Then, the unisexual gametophytes were separated and transferred into individual 20 ml scintillation vials filled with half-strength PES with the addition of 2 ml L^−1^ of a saturated solution of germanium dioxide (6 mg L^−1^) to reduce possible contamination of diatoms (Lewin 1966).

Next, the female and male gametophytes were maintained separately within environmental chambers (Harris Environmental Systems, USA) under red LED conditions or under high output cool white fluorescent lights. Red light conditions were used to prevent the female cultures from producing eggs, or undergoing parthenogenesis, outside of our ideal time frame for crossing because blue light has been linked to being a trigger for such events. Two chambers were controlled with the same 16:8 h light–dark cycle photoperiod with a photon fluence rate adjusted from 20 to 60 μmol photons m^−2^ s^−1^ at 13 °C for more than 12 days. Each culture was fragmented (80–400 μm long filaments) every 30 days over six months by using either dissecting needles, mortars and pestles, or mini blender cups with blades (Redmond et al. 2014).

### Crossing, fertilization, and gametophyte attachment on seedstring

To maximize genetic diversity, crosses were designed to include all original collections and were modified according to the available gametophyte biomass one week before each crossing season. For the 2019–2020 season, male and female gametophytes collected in 2018 from 10 locations were used for making the crosses, and 126 plots were harvested with available data (Supplement Table 1). In the 2020–2021 season, gametophytes from 8 of those locations and from previous farm-grown sporophytes were used to make 106 plots with available data at harvest (Supplement Table 1). All the crosses of gametophytes were made at University of Connecticut (The Seaweed Marine Biotechnology Labs, University of Connecticut, Stamford, CT, USA), starting with the use of micro-scoops, Catalog No. NC0600027 (6–10 mg) (Fisher Scientific). Since it is time-consuming to consistently measure hundreds of gametophytes, micro-scoops were tested and adopted for this breeding program. Each scoop measured 6 mg of male gametophytes wet biomass, and two scoops of female gametophytes were used for makes to keep a 2:1 female:male ratio. The crossing and hatchery preparation for out-planting included several major steps (Supplement Figure 2). To scoop out a certain amount of the gametophytes, a subset of biomass from the gametophyte culturing flasks was collected using sterilized Pasteur pipettes and transferred into prelabeled 1.5 mL centrifuge tubes. To pack the biomass at the bottom of the tubes, we spun them in a centrifuge for 30 s at 21,000 RCF. Then, with the help of the measurement scoops, we collected ~ 6 mg of the male gametophytes and ~ 12 mg of female gametophytes and mixed them together. Next, the mixture was transferred into prefilled flasks to facilitate reproduction and to generate sporophytes at 15 °C with a photon fluence of 80 ± 10 μmol photons m^−2^ s^−1^ in a 12:12 h light–dark cycle. Each cross was checked weekly to estimate the amount and size of juvenile sporophytes under an inverted microscope, model No. CKX 53 (Olympus, Japan).

Once 80% of the juvenile sporophytes within a microscope's field of view at 20X magnification reached 100–300 μm, the crosses were filtered through a 20 μm Nytex mesh (Wildco, USA) to concentrate them into about 1 mL aliquots. While each cross had slightly different numbers of juvenile sporophytes within the 100 mL culturing media, the average number of juvenile sporophytes was about 53 per 1 mL before filtering. Minor losses occurred after filtering. The filtered sporophytes and unquantified associated gametophytes were evenly painted onto 2 mm diameter seedstring pre-wrapped on a PVC puck (6 cm in length and 5 cm in diameter). This was accomplished by spreading a thin layer of the sporophytes/gametophytes mixture (1 mL volume) with a Chinese calligraphy brush (BQLZR, Shenzhen, Guangdong, China). Crosses were painted on 1 m of seedstring, giving us 1 m plots for each individual cross. Then the painted seedstring was covered by 1 mL 0.5% AtSea (AtSeaNovaTechnologies, Ronse, Belgium) according to the manufacturer’s instruction. The painted pucks were aerated to increase the homogeneity of the culture media for about 6 weeks in 250 ml Magenta Plant Culture Boxes (Bio-World, USA) at 15 °C and photon fluence rate of 80 ± 10 μmol photons m^−2^ s^−1^ in a 12:12 h light–dark cycle. Growth media changes were made weekly.

### Outplanting, harvesting, and phenotyping

For both the 2019–2020 and 2020–2021 seasons, crosses were out-planted side-by-side in 1 m intervals at 2.4 m cultivation depth at a common garden farm site in New Castle, New Hampshire, USA (43.0725° N, 70.7161° W). The current speed of the farm was about 1 m s^−1^, with a salinity of 28–33 PSU. The seawater temperature during the growing season ranged from 3.6 °C to 8.4 °C. The pH was about 8.6. The nitrate level from January to May ranged from 1.3—8.7 μM. Planting occurred on December 6, 2019 for the 2019–2020 season, and on December 9, 2020 for the 2020–2021 season. Harvesting for 2019–2020 occurred on June 8, 2020, resulting in 185 growing days. Harvesting for 2020–2021 occurred on June 7, 2021, resulting in 180 growing days.

Each year, sporophytes were harvested and transported in 1800L insulated chilled coolers to the Environmental Systems Laboratory (Woods Hole Oceanographic Institution, MA, USA). Upon arrival, sporophytes were transferred to 1000 L tanks that were supplied with flowing seawater (10℃) prior to phenotyping.

All the harvested plots on the growing ropes were cut into approximately 1 m plots based on crosses’ tags. Each plot was photographed and weighed (wet weight) (Supplement Figure 3). Blade density per plot was estimated by detaching and counting all blades within five randomly predetermined 2 cm long sections per 1 m plot. Five traits of blade morphology (blade length, blade maximum width, blade thickness, stipe length, and stipe diameter), were measured on selected 5 longest mature blades per plot using electronic rulers (eMeasure Inc,, USA) and vernier calipers (Neiko, China).

### Data analysis

Histograms were constructed to summarize measurements of 232 farmed kelp plots harvested in the 2019–2020 and 2020–2021 growing seasons. The distributions with the mean line allowed visual comparisons of the general performance at plot and individual levels for inter- and intra-hybrid plots in each growing season. Correlation matrices were then generated to assess the correlation between plot level traits and individual level traits of two types of crosses, sugar kelp x sugar kelp, and sugar kelp x skinny kelp. Boxplots were constructed to visualize the performance of progeny over the parental sugar and skinny kelps performance. Furthermore, bar plots were created to compare progeny traits with skinny female x sugar male kelp lines, sugar female x skinny male kelp lines, and sugar female x sugar male kelp lines. These comparisons allowed us to determine any maternal or paternal effects of plots with skinny kelp backgrounds. One-way analysis of variance (ANOVA), followed by post-hoc Tukey-HSD multiple comparison tests were used for kelp with different parental types in each growing season. For data that did not meet the parametric assumptions of ANOVA (normality and homoscedasticity), Welch's ANOVA was done to identify which group of crosses behaved differently from each other. When *P* < 0.05, it was considered significant. The analyses depict the visual and statistical differences in parental contributions of different types of crosses to progeny for traits in each season. All the analyses were conducted using R 3.6.3 (R Core Team 2013), and the plots were made with R package ggplot2 (Wickham 2016).

## Results

**Fig. 1:**
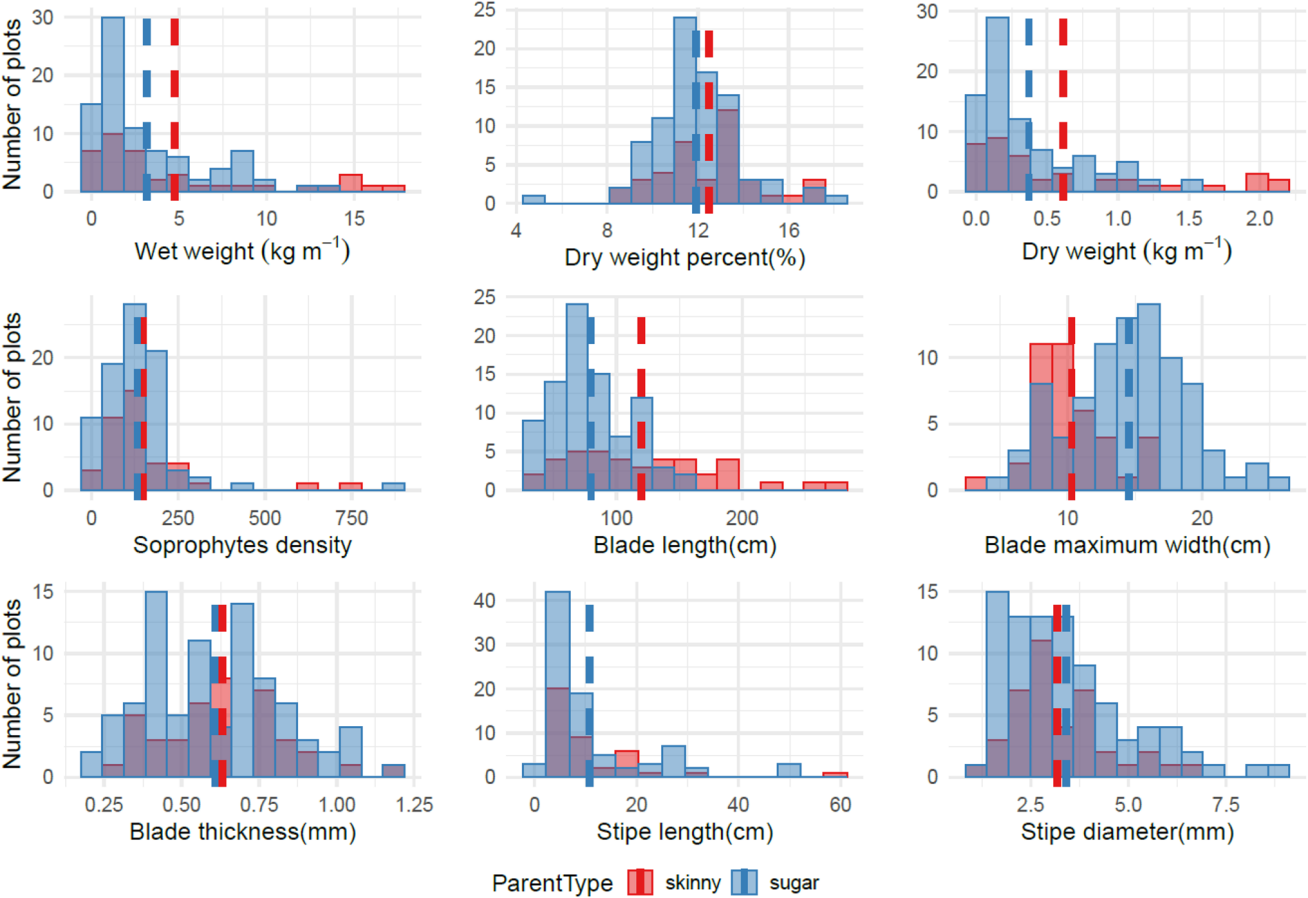
Distribution of all collected phenotypic traits for the 2019–2020 season. The red and blue distribution plots display the crosses related to skinny kelp and pure sugar kelp, respectively, with mean values indicated with dashed lines. For stipe length, the means of the two groups were very similar (sugar: 10.81 cm; skinny: 10.79 cm); only one mean value line was shown here

**Fig. 2:**
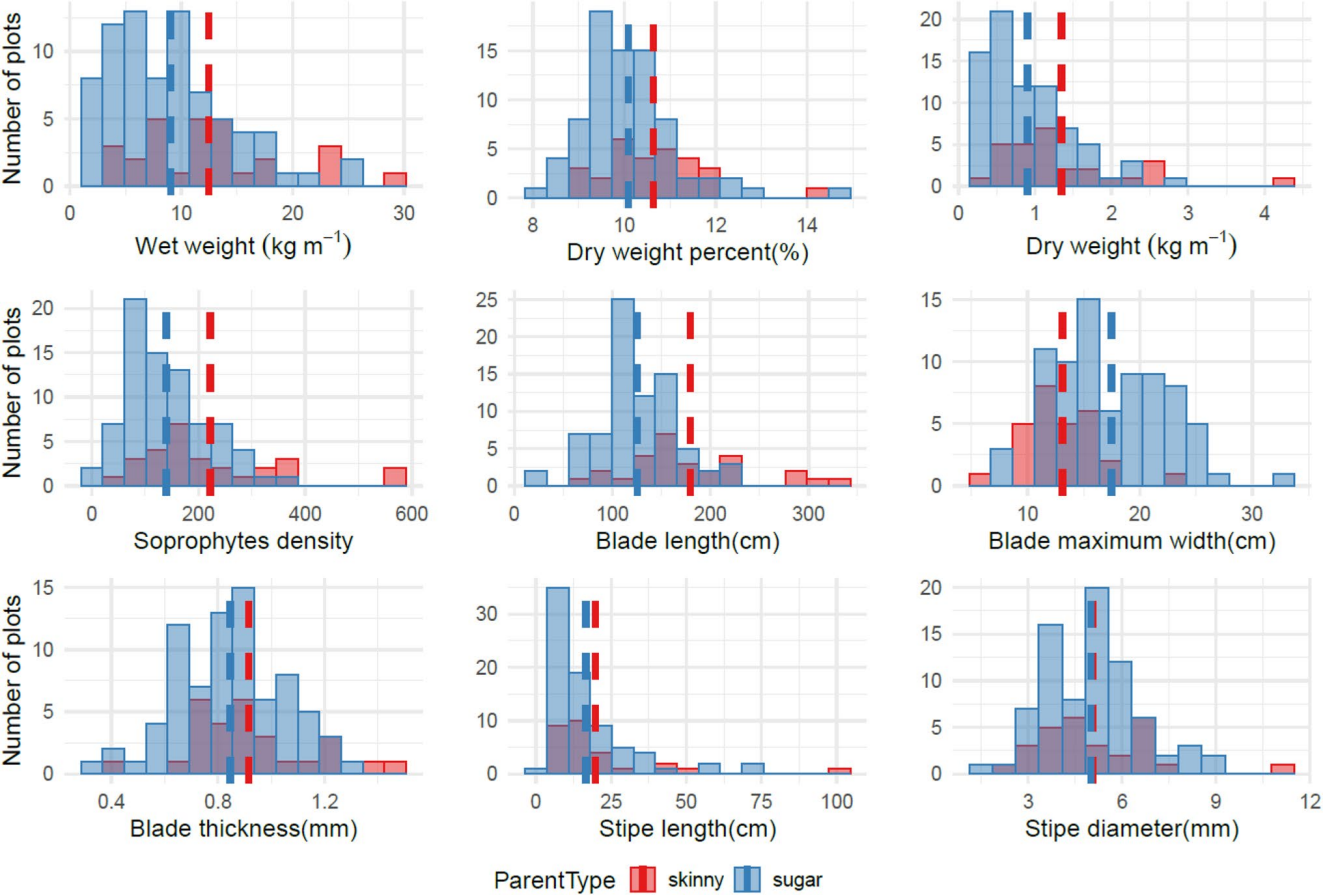
Distribution of all collected phenotypic traits for the 2020–2021 season. The red and blue distribution plots display the crosses related to skinny kelp and pure sugar kelp, respectively, with mean values indicated with dashed lines

Comparisons of 232 plots’ performance distributions between the sugar x sugar and sugar x skinny crosses in 2019–2020 (Fig. 1) and 2020–2021 (Fig. 2) kelp growing seasons showed an overall similar distribution for each trait. For both seasons, the majority of the best performing plots (61% or 8 out of 13 plots) with more than 2 kg m^−1^ dry weight had *S. angustissima* ancestry from either the maternal or paternal side (Figs. 1 and 2). And the plot with a maximum dry weight per meter (> 4 kg m^−1^) in 2020–2021 was a hybrid cross with *S. angustissima*. The morphological trait distributions had similar patterns for the two seasons. The blade length distributions for both seasons showed that kelp plots with skinny kelp ancestry tended to have wider ranges (Figs. 1 and 2). For all plots measured across both farming seasons, 16 plots had blades of more than 2 m in length, from which 75% (12 plots) had *S. angustissima* as a parent. Plots that had *S. angustissima* as a parent tended to have narrower maximum blade width. And the two types of hybrid sporophytes showed similar blade thickness, stipe length, and stipe diameter (Figs. 1 and 2).

**Fig. 3:**
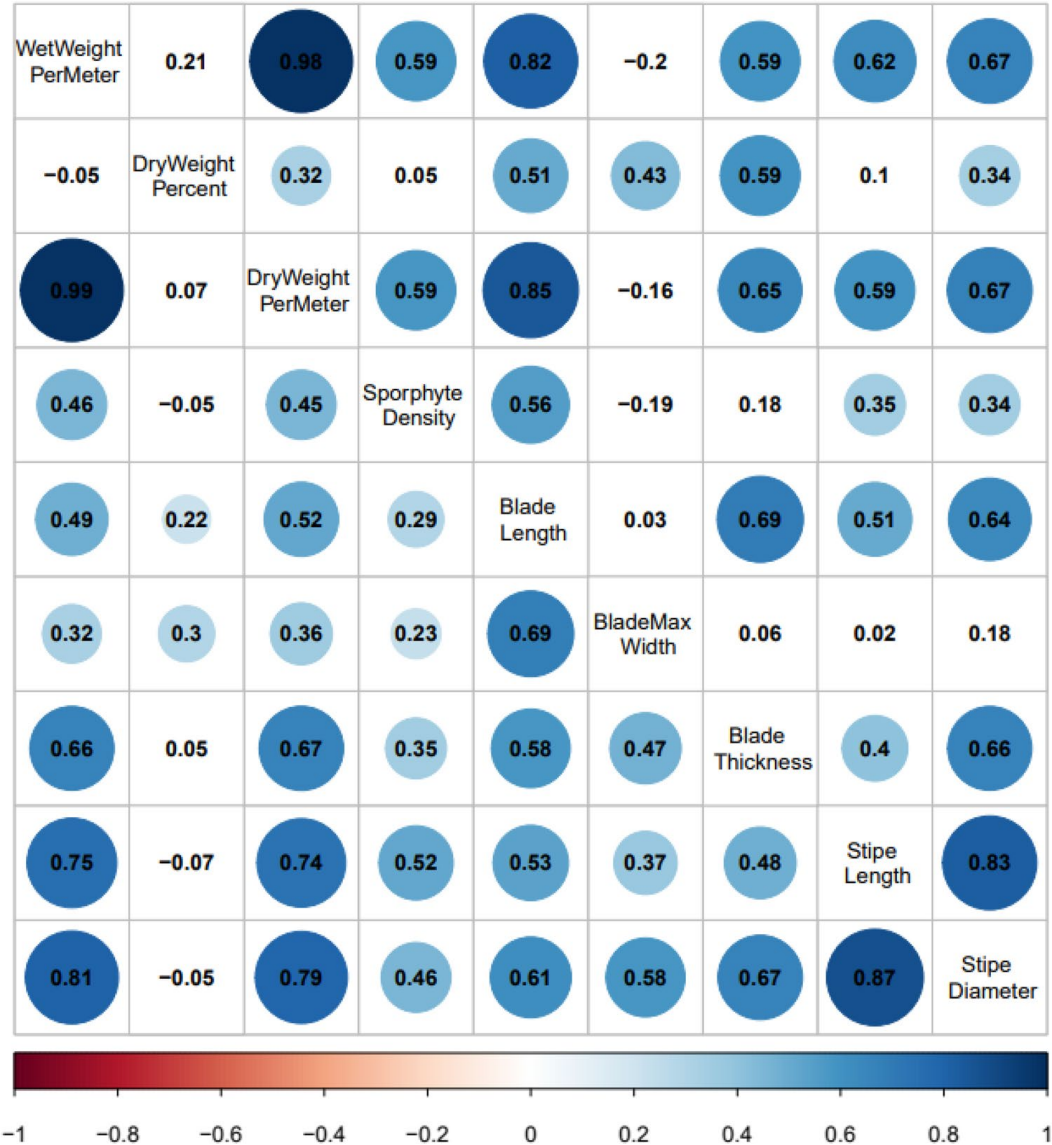
Correlation matrices for the 2019–2020 farm season. Within each season’s correlation matrix, the upper triangle represents the pairwise correlations of collected traits for skinny kelp related progenies (sugar kelp x skinny kelp). In contrast, the lower triangle represents the pairwise correlations of collected traits for only sugar kelp related progenies (sugar kelp x sugar kelp). Numbers represent correlation coefficients between every two variables. Significant correlations (*P* < 0.05) have circles with the circle size indicating the absolute values of correlation coefficients

**Fig. 4:**
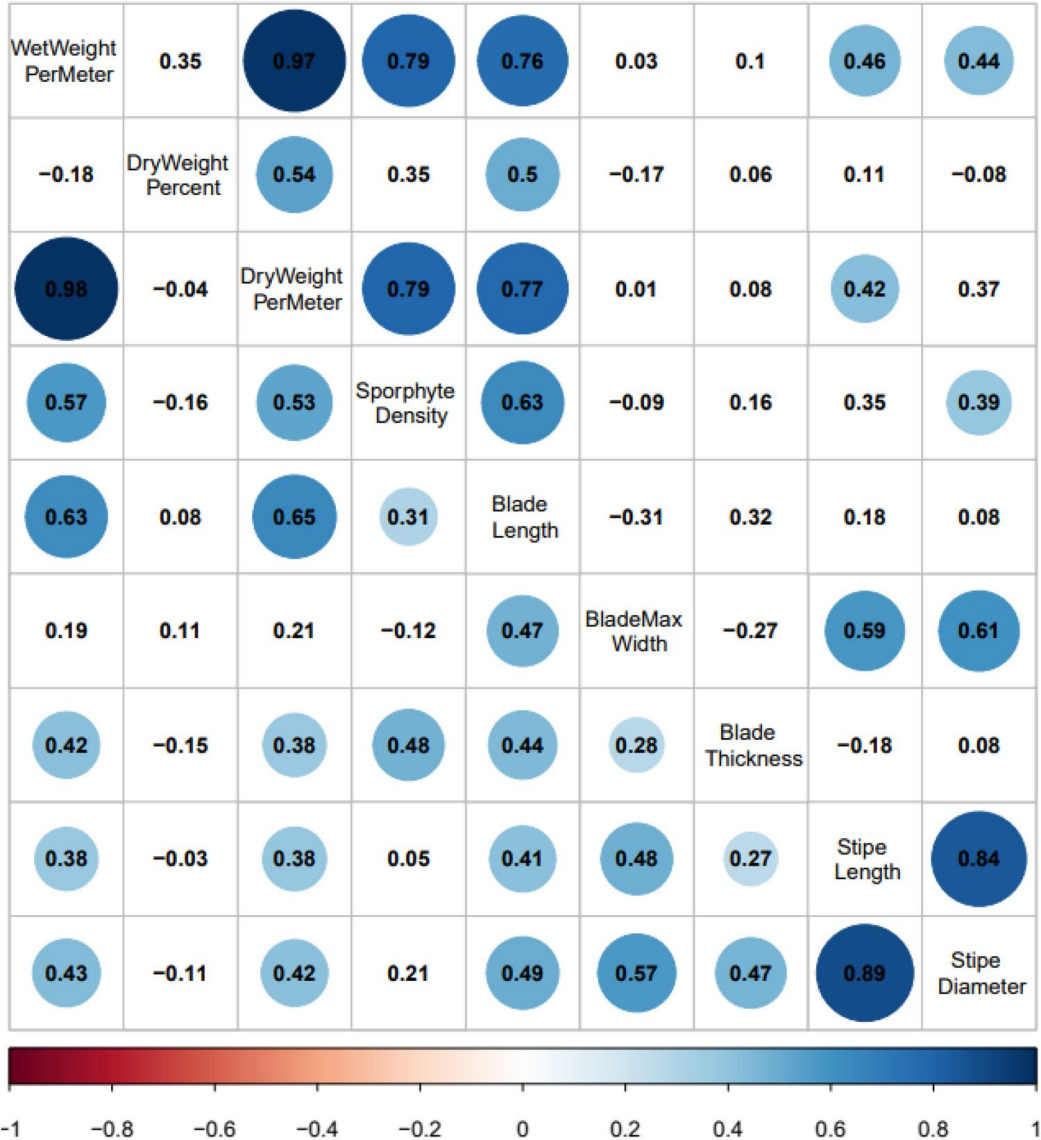
Correlation matrices for the 2020–2021 farm season. Within each season’s correlation matrix, the upper triangle represents the pairwise correlations of collected traits for skinny kelp related progenies (sugar kelp x skinny kelp). In contrast, the lower triangle represents the pairwise correlations of collected traits for only sugar kelp related progenies (sugar kelp x sugar kelp). Numbers represent correlation coefficients between every two variables. Significant correlations (*P* < 0.05) have circles with the circle size indicating the absolute values of correlation coefficients

Pairwise trait correlations indicated that patterns were similar in hybrid plots with (sugar x skinny) and without (sugar x sugar) *Saccharina angustissima* between 2019–2020 and 2020–2021 (Figs. 3 and 4). For both seasons, dry weight per meter for plots with a pure *S. latissima* heritage was positively correlated with wet weight per meter (*P* < 0.05, Figs. 3 and 4), sporophyte density, blade length, blade thickness, stipe length, and stipe diameter. Yet, dry weight per meter was only positively correlated with wet weight per meter, sporophyte density, stipe length, and blade length for plots with at least one *S. angustissima* parent in the 2020–2021 season. Additionally, the dry weight per meter was positively correlated with dry weight percentage for plots with *S. angustissima* heritage in both seasons while not significantly correlated in the plots with pure *S. latissima* heritage. At the individual level for morphological traits, sporophytes with pure *S. latissima* heritage exhibited blade lengths significantly and positively correlated with all other measured blade level traits (*P* < 0.05, Figs. 3 and 4).

**Fig. 5:**
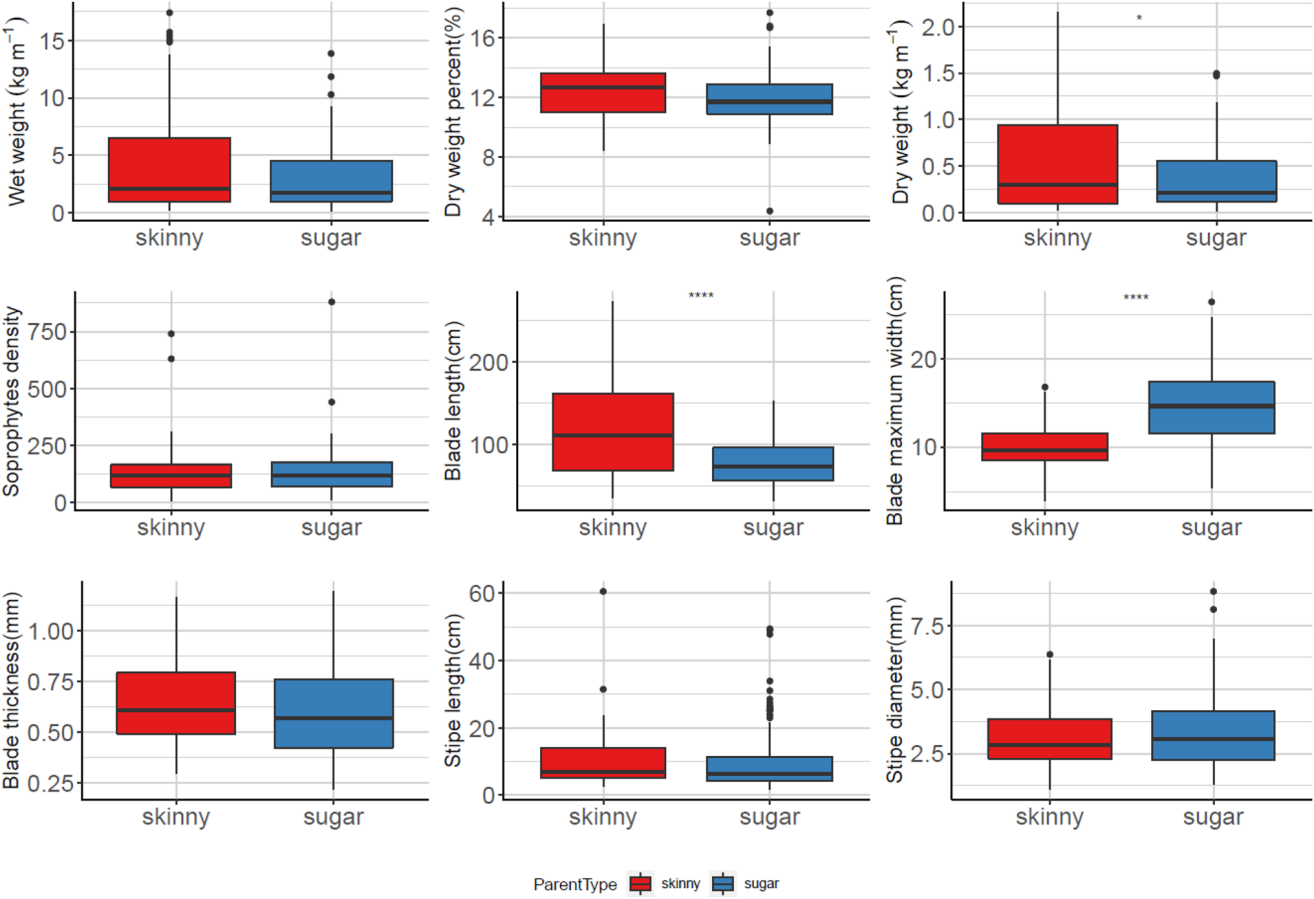
The comparison for skinny kelp related progenies and pure sugar kelp progenies of nine collected traits in 2019–2020 farm seasons. * represents *P* < 0.05;** represents *P* < 0.01; *** represents *P* < 0.001; **** represents *P* < 0.0001 using one-way ANOVA

**Fig. 6:**
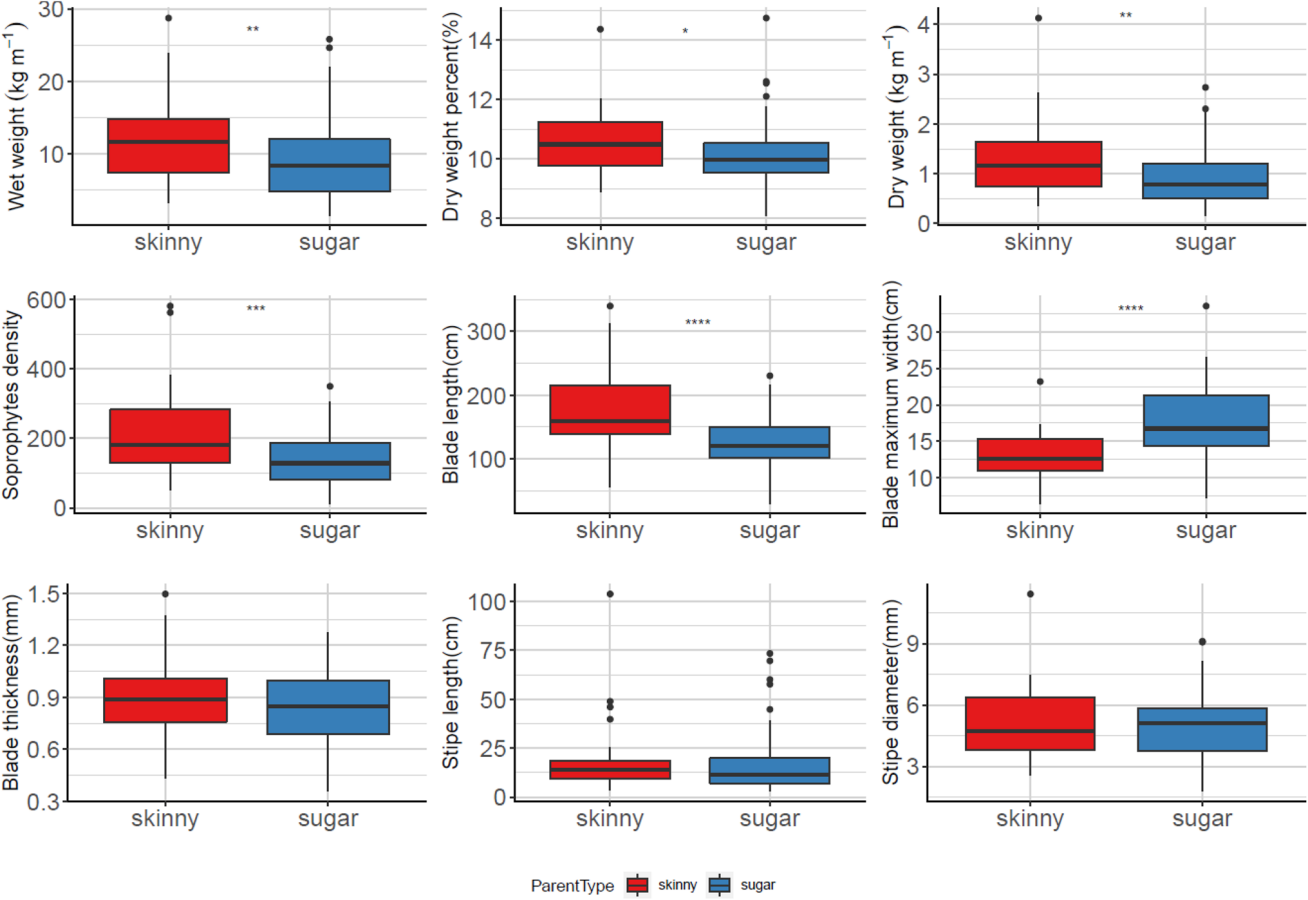
The comparison for skinny kelp related progenies and pure sugar kelp progenies of nine collected traits in 2020–2021 farm seasons. * represents *P* < 0.05;** represents *P* < 0.01; *** represents *P* < 0.001; **** represents *P* < 0.0001 using one-way ANOVA

The comparison of skinny kelp related progenies and pure sugar kelp progenies of nine collected traits showed similar trends in both growing seasons (Figs. 5 and 6). Dry weight per meter, blade length, and blade thickness were significantly different for the two types of progeny plots, indicating that the crosses with skinny kelp parents tended to have a higher dry weight per meter and longer narrower blades. For wet weight per meter, dry weight percentage, and sporophyte density, statistical significance was only detected in the 2020–2021 season, indicating that progenies with a skinny kelp parent had higher wet weight, higher dry weight percentage, and more sporophytes per plot in that growing season (Fig. 6).

**Fig. 7:**
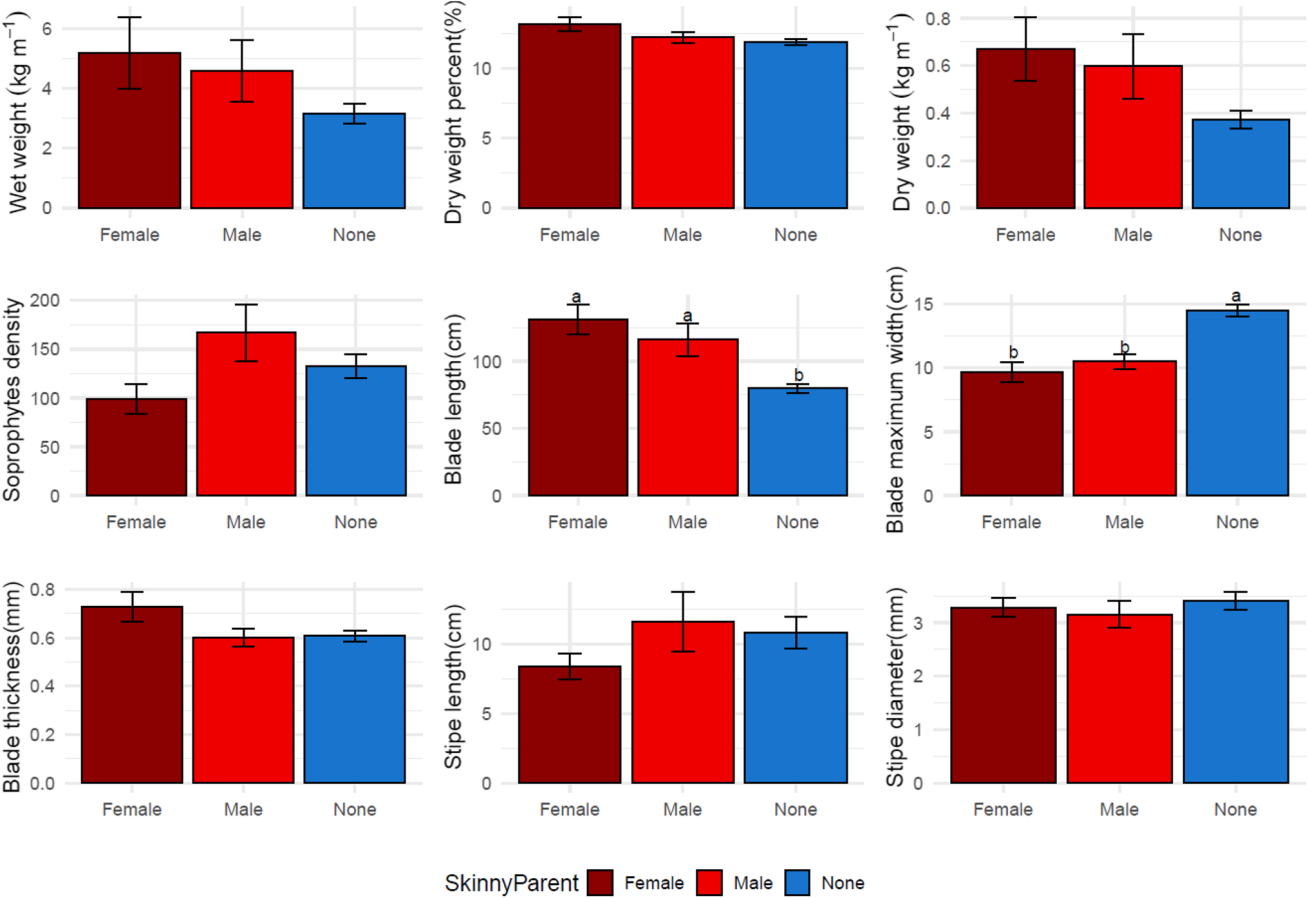
Comparisons between maternal skinny kelp progenies, paternal skinny kelp progenies, and pure sugar kelp progenies of nine collected traits in the 2019–2020 farm season. Different letters indicate a statistically significant difference (*P* < 0.05) among progenies using one-way ANOVA

**Fig. 8:**
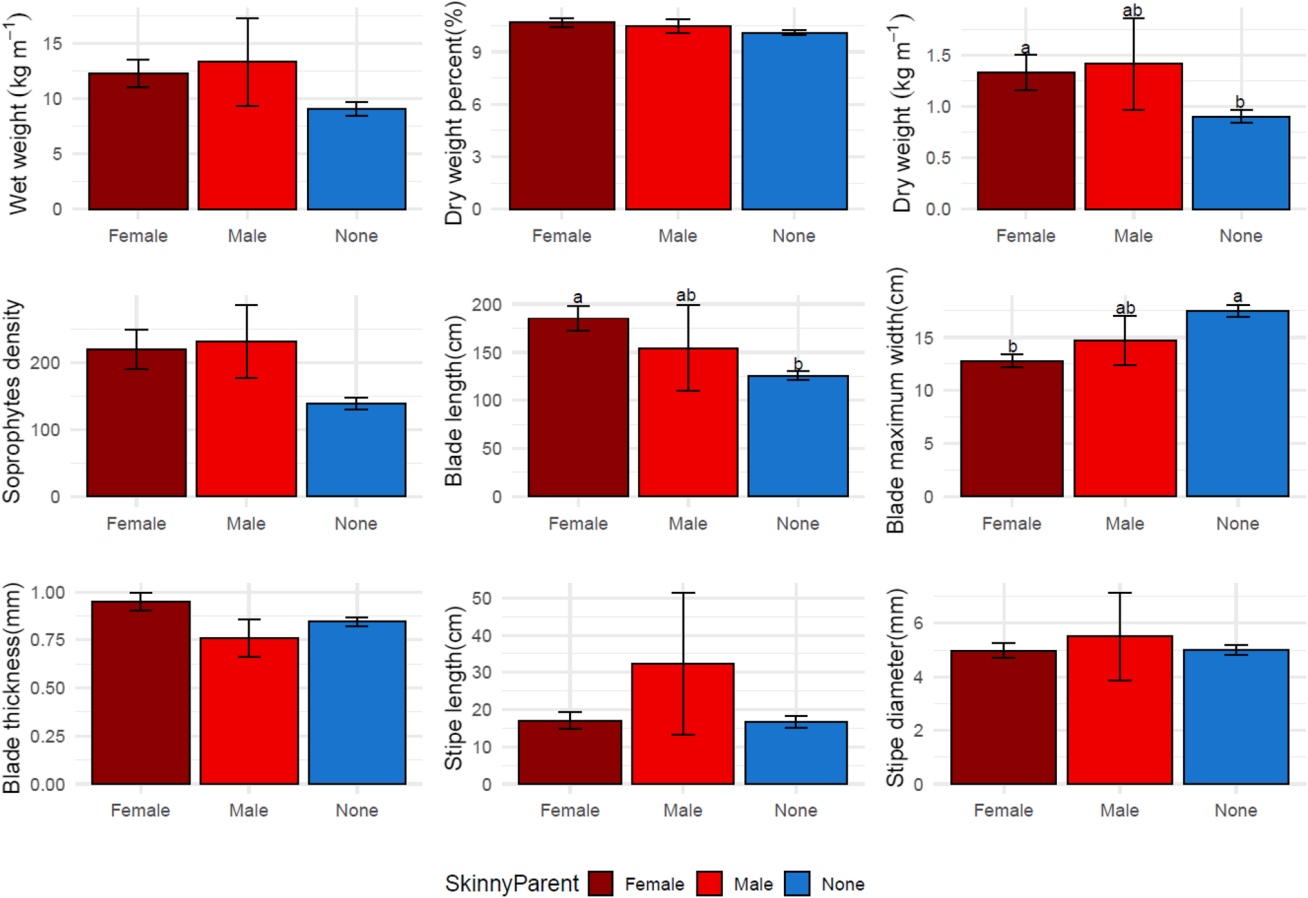
Comparisons between maternal skinny kelp progenies, paternal skinny kelp progenies, and pure sugar kelp progenies of nine collected traits in the 2020–2021 farm season. Different letters indicate a statistically significant difference (*P* < 0.05) among progenies using one-way ANOVA

Assessments of the potential differences between maternal and paternal *S. angustissima* contributions showed that regardless of sex, *S. angustissima* parents contribute to plots with higher dry biomass per meter when compared to plots derived from *S. latissima* only (Fig. 7, Fig. 8). A similar trend was observed for sporophyte density. At the morphological trait level, sporophytes with *S. angustissima* paternal or maternal contribution appeared to have longer and wider blades (Figs. 7 and 8).

## Discussion

Our results indicated that cultivated crosses between *Saccharina* spp*.* produce better farm yields, with higher dry weight per meter (over two seasons) and higher wet weight per meter (in one of those seasons) than crosses of only *S. latissima*. Yield-related traits usually have complex genetic architecture controlled by many "small effect" genes underlying quantitative trait loci and can be affected by various environmental factors in different growing seasons (Huang et al. 2010; Zaw et al. 2019; Cao et al. 2020). Reports from breeding programs in Asia have shown that developing hybrid lines can be an efficient way to increase yield traits in kelp, specially *Saccharina japonica* production (Li et al. 2007, 2008, 2016a, b; Hu et al. 2021). However, crossing sugar kelp with skinny kelp to create hybrid kelp with increased per unit area production has not been previously reported. The influence of farm environmental factors, seedling attaching method, and the plasticity of the hybrid progenies need to be assessed in the future.

In this study, the progeny plots related to *S. angustissima* consistently showed higher dry weight per meter in the two growing seasons (Figs. 5 and 6). Although no statistical significance was detected, a recent study also showed that hybrid plots with *S. angustissima* background tended to have a higher wet weight per meter in the 2018–2019 growing season (Umanzor et al. 2021). These outcomes implied that including *S. angustissima* as a parent would result in farmed kelp progenies with higher yields. It is key to acknowledge that increased yields may be affected by environmental differences (including the length of the growing season) and the particular genetic background of *S. latissima* (Breton et al. 2018; Mao et al. 2020) with which *S. angustissima* is crossed. Therefore, selecting lines using proven methods such as genomic selection (Jannink et al. 2010; Crossa et al. 2017; Huang et al. 2022), advanced phenotyping techniques (Rouphael et al. 2018; Fasoula et al. 2020), and testing in multiple seasons or locations are essential for consistently breeding high yield *S. latissima* crosses.

Despite the previous report that these two species have similar genetic backgrounds (Mao et al. 2020), genes related to increased yield appear in hybrid progenies, and may also be associated with morphological trait variations, as we have seen in this study, where the plots with longer blades tended to have a higher yield (Fig. 3). It will be imperative to collect and keep germplasm seed banks of different strains to ensure the genetic diversity of both *S. latissima* and *S. angustissima* from which to breed and select lines for particular targets, such as high yield and long blades (Wade et al. 2020).

Aside from increasing farm yields, high-yield kelp crosses are expected to provide high-quality gametophytes as "seed" for sustainable use on kelp farms. A gametophyte "seed bank" would prevent overharvesting natural populations in the future. Our two years of data confirmed that crossing *S. latissima* and *S. angustissima* gametophytes can produce hybrid juvenile sporophytes with viable sorus tissue after 4–5 months on our common garden farm site in the Gulf of Maine (Augyte et al. 2017; Umanzor et al. 2021). Meiospores have been successfully germinated to produce a new generation of viable gametophytes in our breeding. In fact, several of the crosses created in the 2020–2021 kelp growing season were produced from gametophytes generated by skinny kelp x sugar kelp hybrid progenies in the 2018–2019 season, which matured to produce viable meiospores. Success in gametophyte and sporophyte viability confirms that farms could be seeded with high-yield hybrid progenies if reproductive tissue was collected from kelp farms. In addition to the original *S. latissima* collections (Mao et al. 2020), our breeding program built and increased more gametophyte collections with various skinny kelp backgrounds. These gametophyte collections are available for farm cultivation, new kelp line breeding, and other future kelp-related genetic research.

In addition to increasing yield, we also found that adding a *S. angustissima* parent changed the morphological traits of the hybrid progenies such as narrower blade width and longer length (Figs. 5 and 6). Breeding for specific morphological traits requires complementary strategies and the combination of genomic and genetic tools with multiple years of phenotyping can accelerate breeding cycles for combining multiple target traits (Valero et al. 2017; Hwang et al. 2019; Hwang and Park 2020; Brakel et al. 2021). Like the yield-related traits, no evidence was found that the morphological traits were linked with just one of the sexes of the parental lines (Figs. 7 and 8). Using *S. angustissima* as either paternal or maternal lines showed similar morphological and higher yield traits in the progenies.

## Conclusion

This study confirmed that *Saccharina latissima* and *Saccharina angustissima* could be successfully crossed to produce mature sporophytes with higher yields and special morphological traits. The hybrid sporophytes that produced sorus tissue had viable meiospores that developed into sexually mature gametophytes. Using skinny kelp and sugar kelp progenies can help determine the genetic regions related to narrow blade traits and further genetic research on kelp breeding. No evidence was found that the narrow blade morphological trait was determined by sex. In the future, we expect to apply these findings to increase the genetic diversity of our gametophyte collections and accelerate the selection and breeding of advanced *Saccharina* crosses.

## Supplementary Information

Below is the link to the electronic supplementary material.

Supplementary file1 (DOCX 1951 kb)

## Data Availability

Data obtained in the experiments herein can be available upon request.
