# Supplementary material for: Skinny kelp (Saccharina angustissima) provides valuable genetics for the biomass improvement of farmed sugar kelp (Saccharina latissima)
Source: J Appl Phycol. 2022 Aug 20;34(5):2551–63. doi: 10.1007/s10811-022-02811-1 (PMC9391627; doi:10.1007/s10811-022-02811-1)
Supplement: Supplementary file 1 — Supplementary file1 (DOCX 1951 kb) [file 10811_2022_2811_MOESM1_ESM.docx]

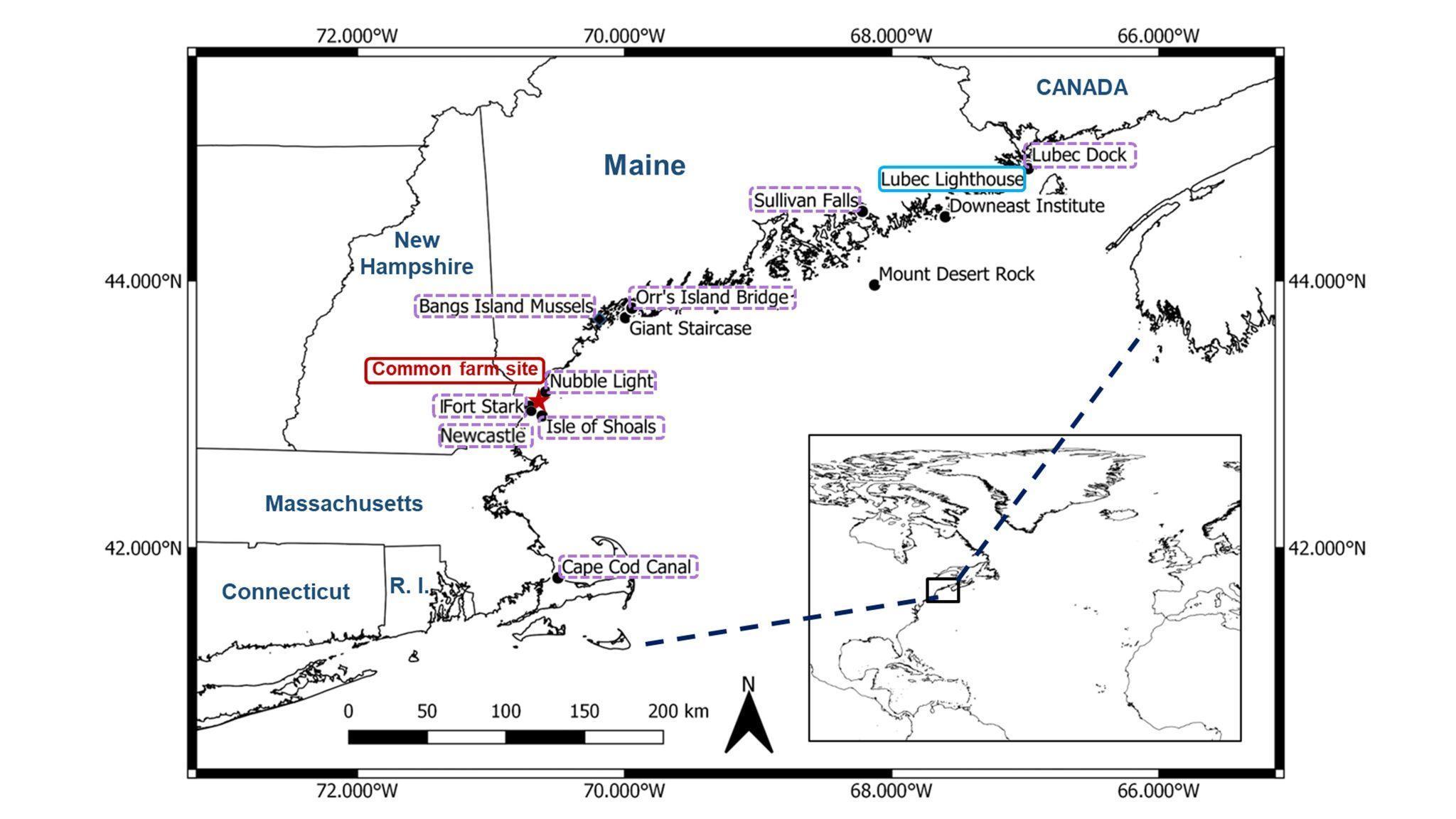


Supplement figure 1 The original locations of kelp sporophytes collected for obtaining the gametophytes used in this study. The original locations of the gametophytes used in the 2019-2020 and 2020-2021 seasons are within the dashed purple squares. The original locations of gametophytes used in only the 2019-2020 season are shown within the s blue square, and the solid red reduare indicates the common farm site.


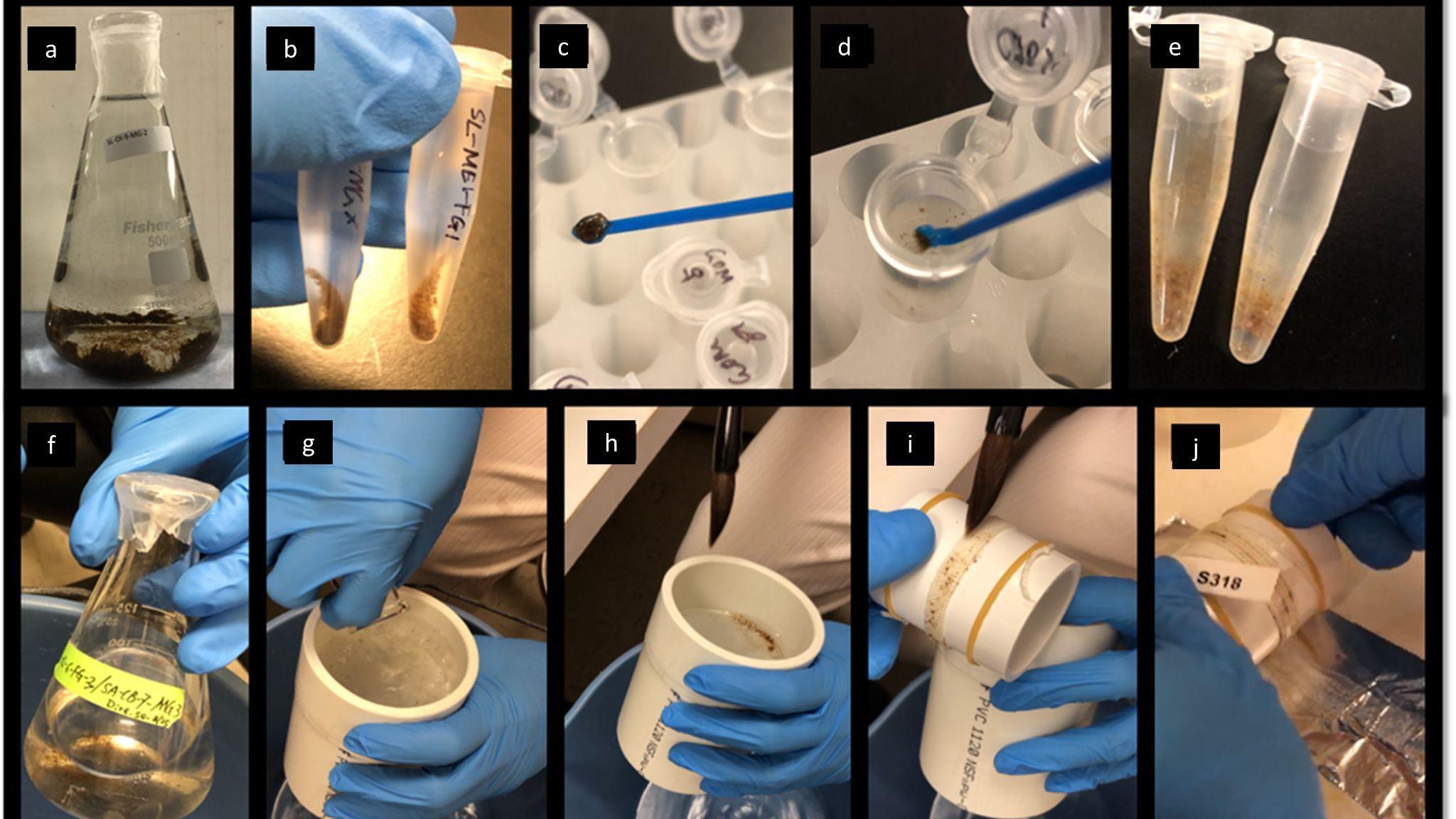


Supplement figure 2 The crossing and painting procedures.


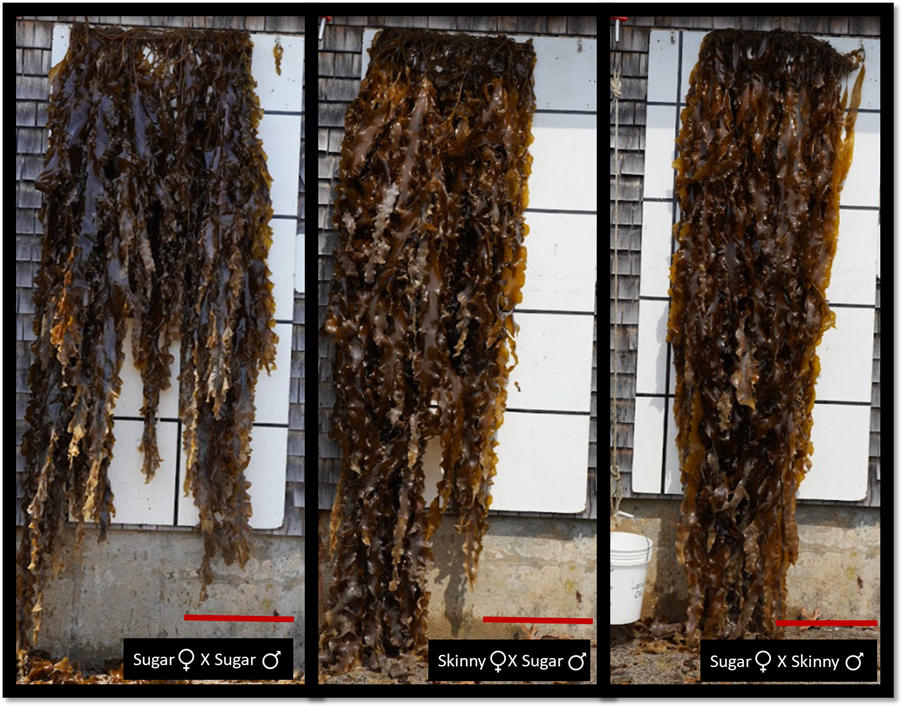
Supplement figure 3 The heaviest wet per meter plot in the 2020-2021 season for three different sugar kelp and skinny kelp crosses. The red line on each plot represents 50 cm.

Supplement Table 1 The crosses in each season with the original collecting locations of female and male parent gametophytes. Casco Bay, ME; Cape Cod, MA; Fort Stark, NH; Lubec Dock, ME; Lubec Lighthouse, ME; Newcastle, NH; Nubble Lighthouse, ME; UNH Outer Docks, NH; Orr’s Island, ME; Sullivan Falls, ME; Casco Bay, ME; Cape Cod, MA; Fort Stark, NH; Lubec Dock, ME; Newcastle, NH; Nubble Lighthouse, ME; UNH Outer Docks, NH; Orr’s Island, ME; UCONN: gametophytes collected from previous farm seasons.

| FarmSeason | Female parent | Male parent | Crosses number | Skinny kelp related |
| --- | --- | --- | --- | --- |
| 2019-2020 | CC | **CB** | 5 | Yes |
| 2019-2020 | JS | **CB** | 5 | Yes |
| 2019-2020 | LD | **CB** | 1 | Yes |
| 2019-2020 | LL | **CB** | 2 | Yes |
| 2019-2020 | NC | **CB** | 2 | Yes |
| 2019-2020 | NL | **CB** | 3 | Yes |
| 2019-2020 | OD | **CB** | 1 | Yes |
| 2019-2020 | OI | **CB** | 6 | Yes |
| 2019-2020 | SF | **CB** | 5 | Yes |
| 2019-2020 | **CB** | CC | 3 | Yes |
| 2019-2020 | **CB** | JS | 3 | Yes |
| 2019-2020 | **CB** | LD | 1 | Yes |
| 2019-2020 | **CB** | NC | 1 | Yes |
| 2019-2020 | **CB** | OI | 2 | Yes |
| 2019-2020 | CC | CC | 2 | No |
| 2019-2020 | JS | CC | 1 | No |
| 2019-2020 | NC | CC | 1 | No |
| 2019-2020 | OD | CC | 1 | No |
| 2019-2020 | OI | CC | 3 | No |
| 2019-2020 | CC | JS | 11 | No |
| 2019-2020 | NC | JS | 2 | No |
| 2019-2020 | OI | JS | 3 | No |
| 2019-2020 | SF | JS | 2 | No |
| 2019-2020 | CC | LD | 1 | No |
| 2019-2020 | JS | LD | 3 | No |
| 2019-2020 | LD | LD | 1 | No |
| 2019-2020 | OI | LD | 1 | No |
| 2019-2020 | CC | LL | 1 | No |
| 2019-2020 | CC | NC | 10 | No |
| 2019-2020 | JS | NC | 1 | No |
| 2019-2020 | NC | NC | 2 | No |
| 2019-2020 | NL | NC | 1 | No |
| 2019-2020 | OI | NC | 2 | No |
| 2019-2020 | SF | NC | 2 | No |
| 2019-2020 | NC | NL | 3 | No |
| 2019-2020 | CC | OD | 2 | No |
| 2019-2020 | JS | OD | 1 | No |
| 2019-2020 | NL | OD | 1 | No |
| 2019-2020 | CC | OI | 15 | No |
| 2019-2020 | JS | OI | 3 | No |
| 2019-2020 | LD | OI | 1 | No |
| 2019-2020 | NC | OI | 3 | No |
| 2019-2020 | NL | OI | 2 | No |
| 2019-2020 | OI | OI | 2 | No |
| 2019-2020 | SF | OI | 1 | No |
| 2019-2020 | LD | SF | 1 | No |
| 2020-2021 | **CB** | JS | 3 | Yes |
| 2020-2021 | **CB** | LD | 4 | Yes |
| 2020-2021 | **CB** | NC | 1 | Yes |
| 2020-2021 | **CB** | NL | 7 | Yes |
| 2020-2021 | **CB** | OI | 3 | Yes |
| 2020-2021 | CC | **CB** | 2 | Yes |
| 2020-2021 | SF | **UCONN (CBxLL)** | 2 | Yes |
| 2020-2021 | **UCONN (CBxLL)** | NC | 4 | Yes |
| 2020-2021 | **UCONN (NLxCB)** | OI | 2 | Yes |
| 2020-2021 | CC | CC | 7 | No |
| 2020-2021 | CC | JS | 1 | No |
| 2020-2021 | CC | NC | 3 | No |
| 2020-2021 | CC | OD | 1 | No |
| 2020-2021 | CC | OI | 1 | No |
| 2020-2021 | CC | UCONN (OIxNL) | 4 | No |
| 2020-2021 | JS | CC | 2 | No |
| 2020-2021 | JS | JS | 4 | No |
| 2020-2021 | JS | LD | 3 | No |
| 2020-2021 | JS | NC | 2 | No |
| 2020-2021 | JS | NL | 2 | No |
| 2020-2021 | JS | OI | 1 | No |
| 2020-2021 | LD | OD | 1 | No |
| 2020-2021 | NC | CC | 2 | No |
| 2020-2021 | NC | NL | 3 | No |
| 2020-2021 | NC | OD | 2 | No |
| 2020-2021 | NC | OI | 1 | No |
| 2020-2021 | NL | OI | 9 | No |
| 2020-2021 | OI | JS | 1 | No |
| 2020-2021 | OI | NC | 16 | No |
| 2020-2021 | SF | NC | 6 | No |
| 2020-2021 | UCONN (OIxNL) | LD | 2 | No |
| 2020-2021 | UCONN (OIxNL) | OI | 2 | No |
| 2020-2021 | UCONN (OIxSF) | OI | 2 | No |
